# Specific Silencing of the REST Target Genes in Insulin-Secreting Cells Uncovers Their Participation in Beta Cell Survival

**DOI:** 10.1371/journal.pone.0045844

**Published:** 2012-09-20

**Authors:** David Martin, Florent Allagnat, Emilie Gesina, Dorothee Caille, Asllan Gjinovci, Gerard Waeber, Paolo Meda, Jacques-Antoine Haefliger

**Affiliations:** 1 Service of Internal Medicine, Centre Hospitalier Universitaire Vaudois, Lausanne, Switzerland; 2 Ecole Polytechnique Fédérale de Lausanne, Faculté des Sciences de la Vie, Lausanne, Switzerland; 3 Department of Cell Physiology and Metabolism, University Medical Center, Geneva, Switzerland; Pennington Biomedical Research Center, United States of America

## Abstract

The absence of the transcriptional repressor RE-1 Silencing Transcription Factor (REST) in insulin-secreting beta cells is a major cue for the specific expression of a large number of genes. These REST target genes were largely ascribed to a function of neurotransmission in a neuronal context, whereas their role in pancreatic beta cells has been poorly explored. To identify their functional significance, we have generated transgenic mice expressing REST in beta cells (RIP-REST mice), and previously discovered that REST target genes are essential to insulin exocytosis. Herein we characterized a novel line of RIP-REST mice featuring diabetes. In diabetic RIP-REST mice, high levels of REST were associated with postnatal beta cell apoptosis, which resulted in gradual beta cell loss and sustained hyperglycemia in adults. Moreover, adenoviral REST transduction in INS-1E cells led to increased cell death under control conditions, and sensitized cells to death induced by cytokines. Screening for REST target genes identified several anti-apoptotic genes bearing the binding motif RE-1 that were downregulated upon REST expression in INS-1E cells, including *Gjd2*, *Mapk8ip1*, *Irs2*, *Ptprn*, and *Cdk5r2*. Decreased levels of Cdk5r2 in beta cells of RIP-REST mice further confirmed that it is controlled by REST, *in vivo*. Using siRNA-mediated knock-down in INS-1E cells, we showed that *Cdk5r2* protects beta cells against cytokines and palmitate-induced apoptosis. Together, these data document that a set of REST target genes, including Cdk5r2, is important for beta cell survival.

## Introduction

Type 1 (T1D) and type 2 (T2D) diabetes are characterized by an absolute or relative insulin deficiency, respectively. In both diseases, loss of functional beta cell mass occurs through beta cell apoptosis [1], [2]. While the triggering events and the nature of the molecular effectors leading to diabetes-associated apoptosis are still disputed, several critical regulators of beta cell survival have been identified (Reviewed in [3]). Importantly, the search for intrinsic pro-survival factors has identified several key proteins, including insulin receptor substrate 2 (IRS2) [4], [5], the anti-apoptotic members of the BCL2 family: BCL2 [6], [7] and BCL2L1 (also called BCLXL) [8], MAPK8IP1 (also called islet brain 1) [9], protein tyrosine phosphatase, receptor type, N (PTPRN, also called islet cell antigen 512) [10], AKT1 (also called AKT/PKB) [11].

Our incomplete knowledge of the mechanisms responsible for the unusual susceptibility of beta cells to metabolic stress and inflammation, imposes that specific positive regulators of beta cell mass are identified. To better understand what is a beta cell, and to attempt improving it under pathological situations [12], we initiated a search for new beta cell-specific genes. Generating transgenic mice expressing the transcriptional repressor REST specifically in beta cells (RIP-REST mice) allowed us to identify the function of a wide group of uncharacterized genes that contains the REST binding motif, called Repressor Element 1 (RE-1) [13]. REST is a zinc finger transcription factor which blocks the expression of neuroendocrine traits in all cell types, but neurons and beta cells. Indeed, REST is commonly absent in mature insulin-producing cells and neurons [14]–[16], but suppresses elsewhere the expression of a large set of RE-1-containing genes, thereby ensuring that their expression is specific to neuroendocrine cell types. Upon ectopic REST expression in the RIP-REST transgenic mice, REST target genes were specifically silenced in beta cells. The resulting phenotype showed that, *in vivo*, RE-1-containing genes are crucial for proper beta cell function [13]. The identification that several *bona fide* REST target genes code for proteins that are key to exocytosis further substantiated our observations [13]. Here, we report the characterization of a novel line of RIP-REST founder mice which demonstrates that RE-1-containing genes are also essential to beta cell survival. These mice featured diabetes as a consequence of a gradual but extensive loss of beta cells through apoptosis. *In vitro* experiments with INS-1E cells transduced with *REST*-expressing adenoviral vectors led to the identification of several new REST target genes, with previous identified role in cell survival, including *Irs2*, *Ptprn*, and CDK5 activator subunit 2 (*Cdk5r2*, also called *p39*). siRNA-specific down-regulation of *Cdk5r2* increased the susceptibility of INS-1E cells to major beta cell death effectors cytokines and palmitate, indicating that this activator of CDK5 has an anti-apoptotic activity in beta cells.

## Results

### Diabetic RIP-REST mice feature hyperglycemia and altered insulin secretion

**Figure 1:**
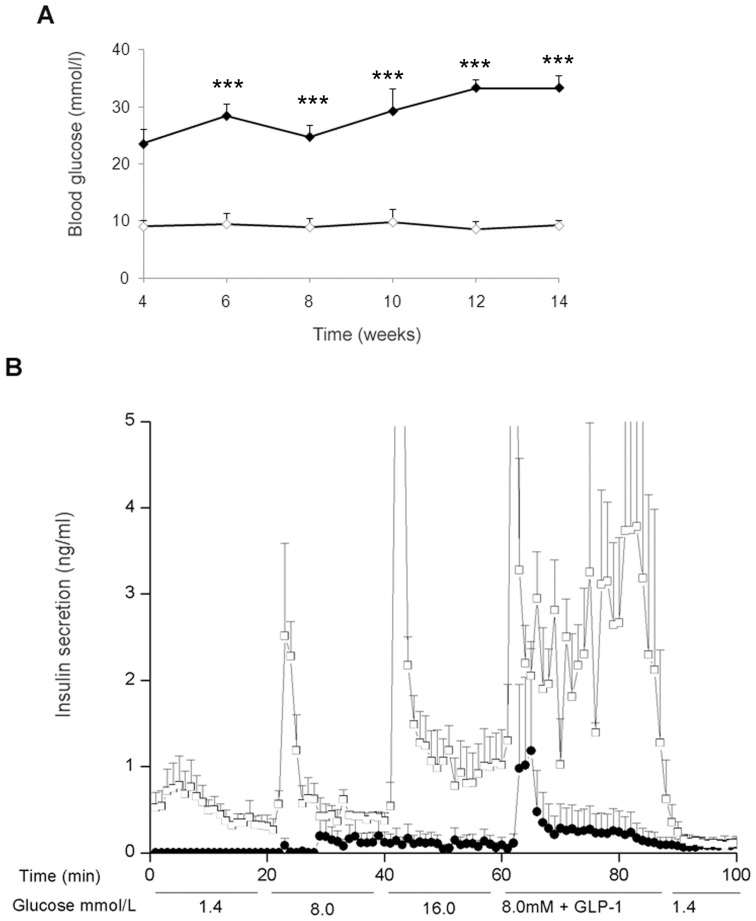
Diabetic RIP-REST transgenic mice are hyperglycemic and show poor insulin secretion. *A*. Blood glucose levels were assessed at different ages in male and female diabetic RIP-REST transgenic (dark diamonds; n = 7) and wild type mice (open diamonds; n = 5). Diabetic RIP-REST mice feature hyperglycemia from weaning onward. Results are mean ± SD. ***P<0.001 versus values of wild type mice. *B*. Diabetic RIP-REST mice (black circles, n = 3) and wild type littermates (open squares, n = 4) were subjected to *in situ* pancreatic perfusion at 1.5 ml/min rate. After a 30-min equilibration period at basal 1.4 mmol/l glucose, the pancreas was perfused sequentially at different glucose concentrations, first at 1.4 mmol/l for 20 min, next at 8.0 mmol/l for 20 min, then at 16.0 mmol/l for 20 min, followed by a 30-min stimulation at 8.0 mmol/l plus 1 nmol/l GLP-1, and finally at 1.4 mmol/l for 15 min. Results are mean ± SD.

We previously reported the characterization of a glucose-intolerant mouse line (referred to as RIP-REST) featuring defects in insulin secretion as well as decreased insulin production, without detectable transgene expression in hypothalamus [13]. Mice from a novel line (referred to as diabetic RIP-REST) showed frank diabetes independently of the gender. These mice featured a glycemia of 23.6±2.6 mmol/l at 4 weeks of age, which increased at 3 month-old up to 33 mmol/l, resulting in lethality after few months. Wild type littermates had a basal glucose level of 9.2±1 mmol/l (Fig. 1A). To assess insulin secretion, the pancreas of 4–5 month-old transgenic and wild type animals were perfused *in situ*. Infusion of 8.0 and 16.0 mmol/l glucose led to a typical biphasic response of insulin secretion in control mice. In contrast, the secretion of the hormone in response to glucose was barely detectable in the diabetic animals (Fig. 1B). The addition of 1 nmol/l GLIP-1 to 8.0 mmol/l glucose largely potentiated insulin release from wild type mice, and to a much lesser extent also in diabetic RIP-REST mice (Fig. 1B).

### Altered insulin secretion of diabetic mice results from massive loss of beta cells

**Figure 2:**
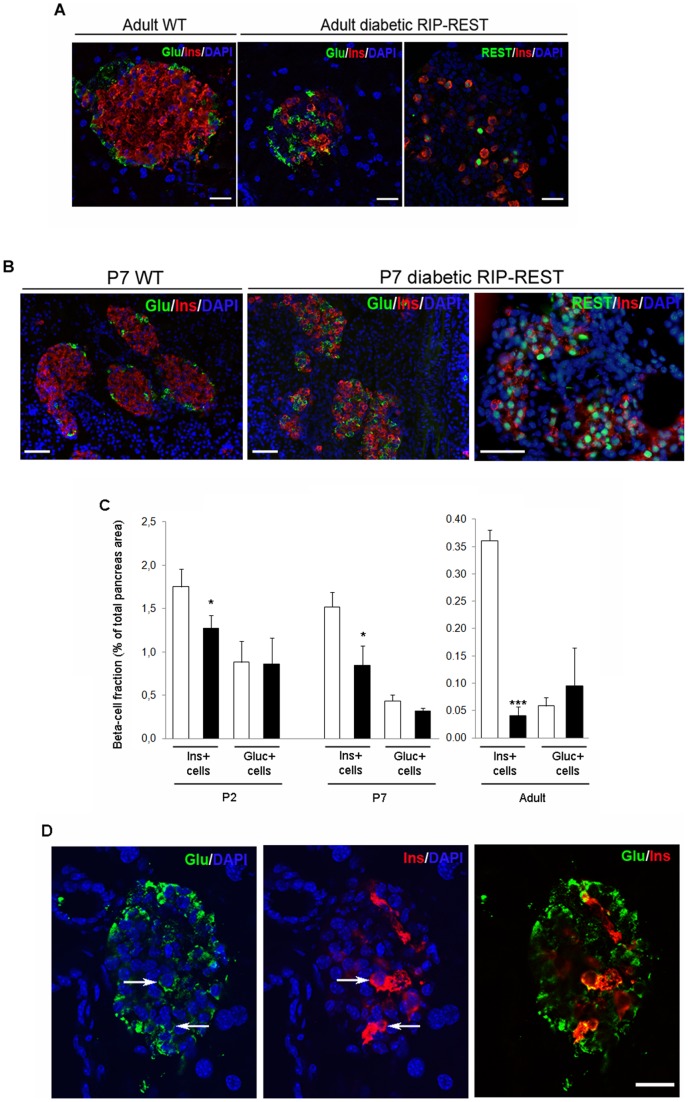
*REST* expression is associated with a major loss of beta cell mass. *A*. Immunostaining for insulin (Ins, red) and glucagon (Glu, green) reveals, when compared to wild type mice (WT, left panel), a major loss of insulin-positive cells, within a low number of disorganized islets in pancreas of adult diabetic RIP-REST mice (middle panel). REST nuclear staining (green, right panel) indicates that only a few surviving beta cells still express REST. Blue staining is the DAPI labeling of nuclei. Scale bar, 25 μm. *B*. Immunostaining for insulin (Ins, red) and glucagon (Glu, green) shows that, even if disorganized when compared to islets from wild type animals (WT, left panel), islets from diabetic RIP-REST mice at postnatal day 7 (P7) show a significant number of insulin-positive cells (middle panel). REST nuclear staining (green, right panel) indicates that a majority of beta cells express REST. Blue staining is the DAPI labeling of nuclei. Scale bar, 50 μm. *C*. Quantification of beta-(Ins+ cells) and alpha- (Gluc+ cells) cell mass in P2, P7 and adult diabetic RIP-REST (black bars, n = 3 each) and wild type mice (white bars, n = 3 each). A 30, 45 and 90% reduction in beta cell mass is observed in P2, P7, and adult diabetic RIP-REST mice, respectively, when compared with corresponding mass of controls. Results are mean ± SD. *P<0.05, ***P<0.001 versus values of wild type mice. *D*. Confocal analysis of insulin (Ins, red) and glucagon (Glu, green) immunofluorescence shows scarce double insulin-and glucagon-expressing cells (arrows) in pancreatic sections of adult diabetic RIP-REST mice. Blue staining is the DAPI labeling of nuclei. Scale bar, 25 μm.

Immunofluorescent labeling of glucagon and insulin in pancreas of 6 month-old mice showed that wild type islets featured, as expected, numerous central beta cells and lower number of alpha cells at the periphery (Fig. 2A left panel). In contrast, those of diabetic RIP-REST mice had much fewer beta cells, and displayed glucagon-positive cells scattered throughout the islets (Fig. 2A middle panel). Strikingly, only a small fraction of the surviving beta cells expressed the *REST* transgene (Fig. 2A right panel). When the same staining was performed on pancreas of 7 day-old mice (P7), a significant number of insulin positive cells was observed in the diabetic mice (Fig. 2B middle panel), of which most expressed the *REST* transgene (Fig. 2B right panel). However, and in contrast to controls (Fig. 2B left panel), alpha cells were already distributed throughout the islets of diabetic mice (Fig. 2B middle panel). To further characterize the time course of the beta cell loss occurring in diabetic mice, we quantified beta- and alpha-cell mass in P2, P7 and adult animals. Compared to wild type mice, we observed a gradual decrease in the beta cell fraction, which was 30 (p<0.05), 45 (p<0.05), and 90% (p<0.001) in P2, P7 and adult diabetic mice, respectively. In contrast, the alpha-cell mass was statistically similar to that of controls at all ages (Fig. 2C). Confocal microscopy also revealed the infrequent occurrence of double insulin- and glucagon-positive cells in adult diabetic RIP-REST mice, which was not observed in wild type mice (Fig. 2D).

### REST expression is associated with beta cell loss due to apoptosis

**Figure 3:**
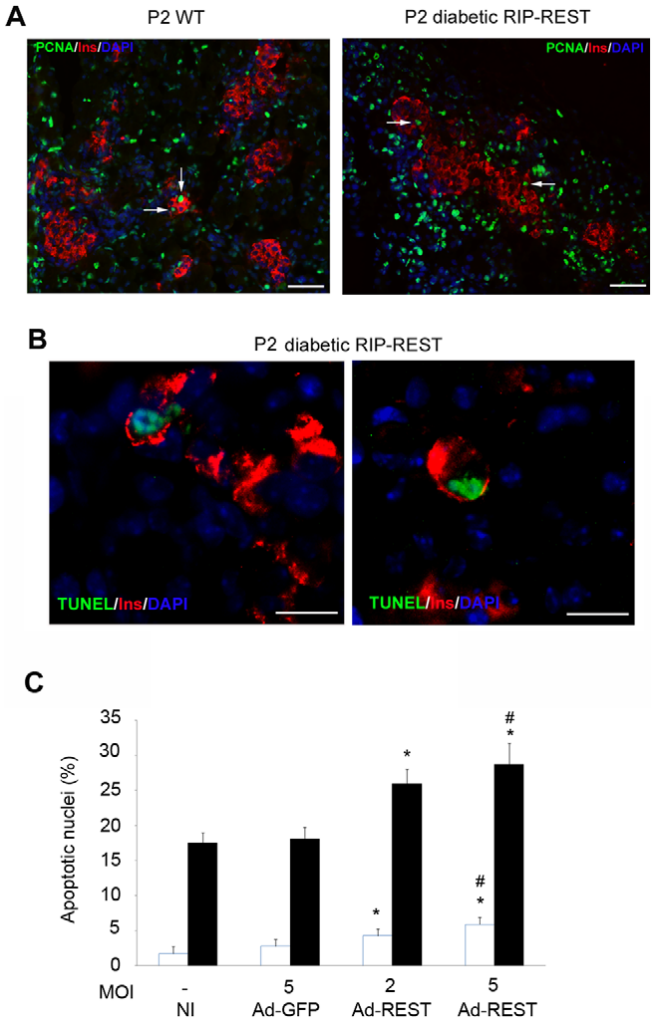
Beta cell loss in diabetic RIP-REST mice occurs through apoptosis. *A*. Nuclear PCNA (green) and insulin (red) staining show same level of proliferating beta cells (arrows) in both wild type (WT, left panel) and diabetic RIP-REST mice (right panel) at P2. Blue staining is the DAPI labeling of nuclei. Scale bar, 50 μm. *B*. Representative images of nuclear TUNEL (green) and insulin (red) staining showing apoptotic nuclei of beta cells in sections of P2 diabetic RIP-REST mice. No apoptotic beta cells were detected in wild type animals (not shown). Blue staining is the DAPI labeling of nuclei. Scale bar, 25 μm. C. Quantification of apoptotic nuclei in non-infected INS-1E cells (NI) and INS-1E cells transfected with a control (Ad-GFP) or REST-expressing adenovirus (Ad-REST) at different multiplicity of infection (MOI) as indicated, and treated 24 h with a mix of cytokines (black bars) or not (white bars). Results are mean ± SD of six independent experiments. *P<0.05 versus respective controls in treated or untreated conditions. # *P*<0.05 versus MOI 2 of Ad-REST infection.

To investigate the postnatal beta cell loss in diabetic RIP-REST mice, we first examined beta cell proliferation by PCNA immunostaining in P2 pancreas. The same proportion of beta cells was labeled for this marker of cell proliferation in wild type (4.9%±1.1; n = 1598), and transgenic mice (6.2%±0.26; n = 833) (Fig. 3A). We next assessed beta cell apoptosis using TUNEL experiments. Whereas no apoptotic beta cell was observed in wild type mice, we detected several in islets of diabetic RIP-REST mice at P2 (Fig. 3B). We did not quantify the proportion of apoptosis, *in vivo*, because apoptotic cells are rapidly cleared by the immune system [17], [18]. However, we assessed apoptosis in INS-1E cells transduced with REST- and GFP-expressing adenoviruses and observed that REST expression *per se* led to an increase in cell death under normal conditions and in a dose-dependent manner (Fig. 3C). The susceptibility of these cells to undergo apoptosis was also increased when cell death was triggered by cytokines (Fig. 3C). Together with the quantifications made in Fig. 2C, these observations suggest that diabetes of transgenic mice was accounted for by postnatal apoptosis of beta cells.

### The levels of REST expression inversely correlate with pancreatic insulin content in RIP-REST transgenic mice

**Figure 4:**
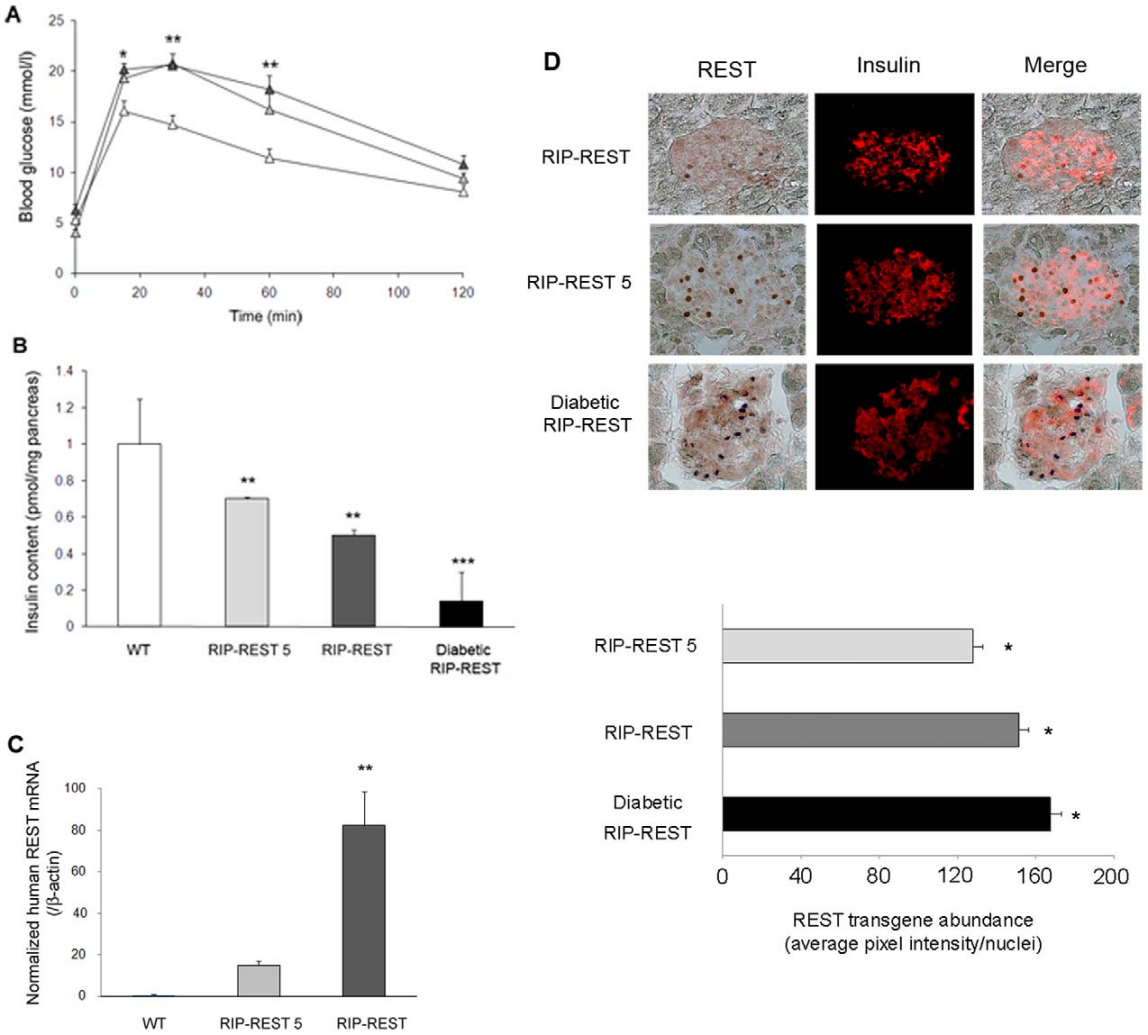
Increasing REST levels in beta cells lead to worsened glucose homeostasis. *A*. Blood glucose levels in 6 month-old wild type (open triangles; n = 8), RIP-REST 5 (gray triangles; n = 8) and RIP-REST males (dark triangles; n = 10) during an IPGTT. After a 14 h fasting, blood samples were taken before (t = 0) and after (t = 15, 30, 60 and 120 min) intraperitoneal injection of glucose (2 g/kg). Results are mean ± SD. *P<0.05, **P<0.01. *B*. Insulin content in pancreas of 5 month-old RIP-REST 5 (light gray bar; n = 8), RIP-REST (dark gray bar; n = 6) and diabetic RIP-REST (black bar; n = 6) transgenic mice reveal a 30, 50 and 85% reduction, respectively, when compared with wild type mice (white bars, n = 6). Results are mean ± SD. **P<0.01, ***P<0.001 versus values of wild type animals. *C*. Quantitative RT-PCR analysis of human *REST* mRNA levels in islets of 5 month-old WT (white bar; n = 5), RIP-REST 5 (light gray bar; n = 6) and RIP-REST (dark gray bar; n = 6) mice. *REST* mRNA levels are six-fold higher in RIP-REST than in RIP-REST 5 animals. Results are mean ± SD. **P<0.01 versus values of wild type animals. *D*. REST transgene abundance in islets from P2 RIP-REST 5, RIP-REST and diabetic RIP-REST mice. Upper panel shows representative images of REST protein levels revealed using specific antibodies against REST and AEC staining of peroxydase activity (nuclear black dots). Parallel immunostaining of insulin (red) and the merge shows colocalization of the two signals. Scale bar, 25 μm. Lower panel shows the quantification of the corresponding average pixel intensity per nuclei for each group. *P<0.05 versus the two other groups.

We analyzed the mice of a third transgenic line (referred to as RIP-REST 5), which was obtained after the pronuclear injection of the RIP-REST transgene. As the other RIP-REST mice, these mice also featured glucose intolerance, as observed by IPGTT (Fig. 4A). Measurement of pancreatic insulin content in each three transgenic line indicated that RIP-REST 5 mice featured a mild 30% decrease in insulin content, whereas RIP-REST mice had a 50% decrease, and diabetic RIP-REST mice a drastic 85% reduction, as compared to wild type mice (Fig. 4B). qPCR analysis of islet mRNA indicated that the expression of the *REST* transgene was six-fold higher in RIP-REST than in RIP-REST 5 mice (Fig. 4C). Diabetic RIP-REST mice could not be investigated by qPCR because of the low number of residual islets. Therefore, REST protein abundance in the three mouse lines was evaluated by semi-quantitative peroxydase REST immunostaining (Fig. 4D upper panel). After image processing, quantification of the average pixel intensity per nuclei revealed that REST abundance was significantly higher in beta cells from diabetic RIP-REST than from RIP-REST mice, itself being higher than in beta cells from RIP-REST 5 mice (Fig. 4D lower panel). These data demonstrate an inverse relationship between the levels of *REST* expression and insulin production, and suggest that high levels of REST in beta cells were sufficient to induce diabetes in transgenic mice.

### RE-1 database screening identifies several RE-1-containing genes involved in beta cell survival

**Figure 5:**
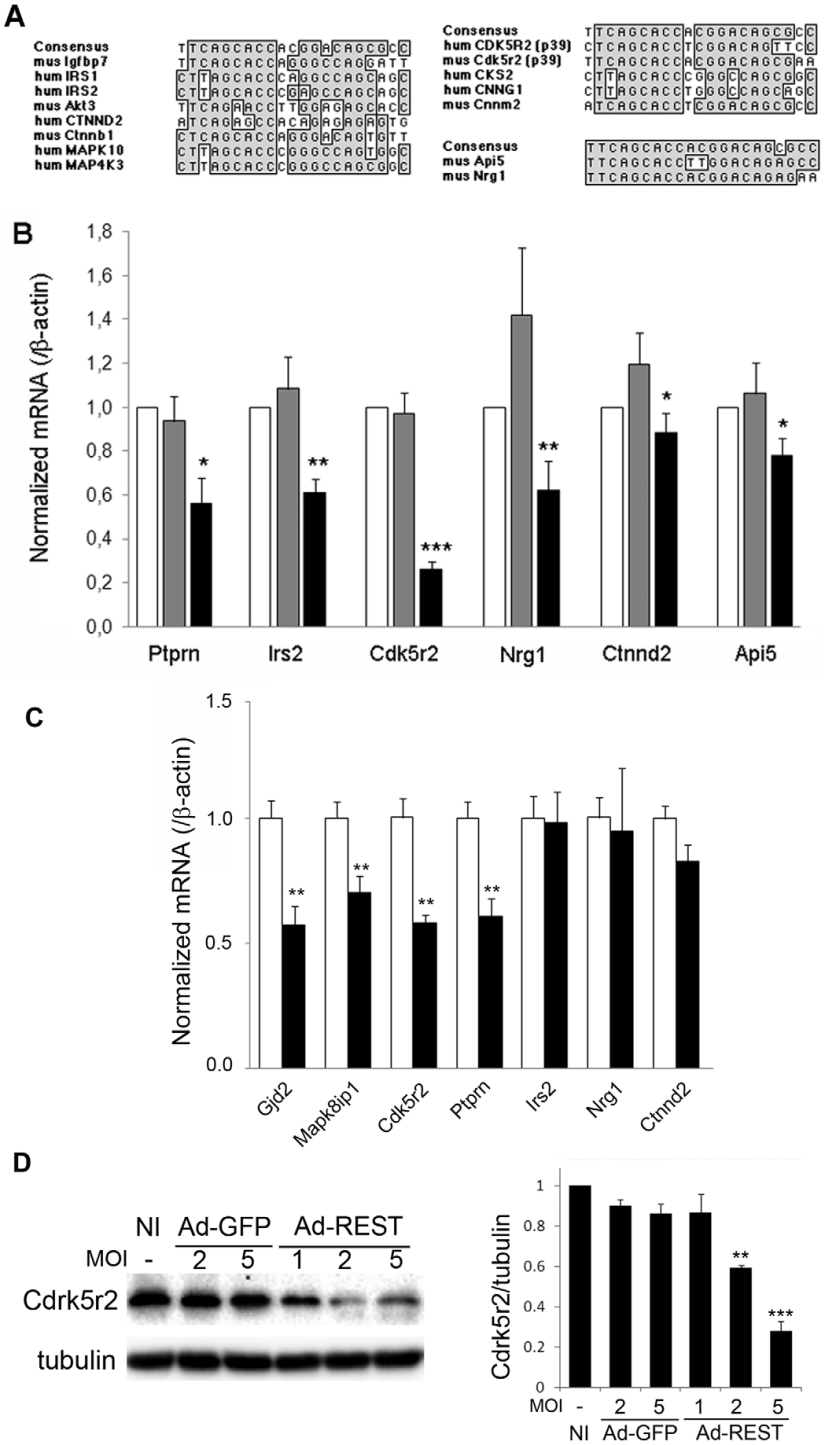
Identification of RE-1-containing genes potentially involved in beta cell survival. *A*. Alignment of identified RE-1 sequences in human (hum) or murine (mus) genes with the consensus RE-1. Left alignment identifies members of the insulin-like growth factor transduction pathway: *Igfbp7*: insulin-like growth factor binding protein 7; *IRS*: insulin receptor substrate; *Akt3*: PKB gamma; *CTNND2*: delta2 catenin; *Ctnnb1*: beta catenin; *MAPK10*: JNK3; *MAP4K3*: MEK kinase kinase 3. Upper right alignment identifies members of the cyclin-related family of mitogenic factors: *Cdk5r2*: cyclin-dependent kinase 5 regulatory subunit 2; *CKS2*: CDC28 protein kinase regulatory subunit 2; *CNNG1*: cyclin G1; *Cnnm2*: cyclin M2. Lower right alignment identifies *Api5*: apoptosis inhibitor 5 and *Nrg1*: neuregulin1. *B*. Quantitative RT-PCR analysis of mRNA levels from control INS-1E cells (white bars) and INS-1E cells infected with GFP-expressing adenovirus (gray bars) or *REST*-expressing adenovirus (black bars). *Ptprn*: protein tyrosine phosphatase, receptor type, N; *Irs2*: insulin receptor substrate2; *Cdk5r2*: cyclin-dependent kinase 5 regulatory subunit 2; *Nrg1*: neuregulin1; *Ctnnd2*: delta2 catenin; *Api5*: apoptosis inhibitor 5. Results are mean ± SD of six independent experiments. *P<0.05, **P<0.01, ***P<0.001 versus INS-1E cells transduced with GFP. *C*. Quantitative RT-PCR analysis of mRNA levels from islets of WT (white bars) (n = 5) and RIP-REST (black bars) (n = 5) mice. *Gjd2*: Connexin36; *Mapk8ip1*: Ib1; *Cdk5r2*: cyclin-dependent kinase 5 regulatory subunit 2; *Ptprn*: protein tyrosine phosphatase, receptor type, N; *Irs2*: insulin receptor substrate2; *Nrg1*: neuregulin1; *Ctnnd2*: delta2 catenin. Results are mean ± s.e.m. **P<0.01 versus values of wild type animals. D. Left panel, immunoblotting of total proteins from non infected (NI) and infected (Ad-GFP, Ad-REST) INS-1E cells showing decreased protein levels of Cdk5r2 upon REST expression. The right panel shows the corresponding quantifications of REST protein levels in INS-1E cells after infection. Data are mean ± SEM of 4 independent experiments. **P<0.01, ***P<0.001 versus the respective Ad-GFP-infected and NI conditions.

We screened a RE-1 database (http://www.bioinformatics.leeds.ac.uk/cgi-bin/RE1db/nrse.cgi) [19] to search for RE-1-containing genes thought to be involved in neuroendocrine-cell protection. We searched genes with anti-apoptotic properties and a conserved RE-1 sequence. We identified 14 RE-1-containing genes that may be involved in growth signals transduction pathways (including members of the insulin-like growth factors pathway, AKT1, and catenin isoforms), in transduction of apoptosis/proliferation (including mitogen-activated protein kinases) and in downstream mechanisms of mitogenesis (including cyclins, cyclin-dependent kinase activators) (Fig. 5A).

We then used qPCR analysis to evaluate the expression of the corresponding mRNAs upon REST expression in INS-1E cells. These experiments showed that the majority of the selected candidates were not transcriptionally regulated by REST (data not shown). However, the expression of the genes coding for IRS2, PTPRN, CDK5R2, neuregulin1 (NRG1), delta2 catenin (CTNND2) and apoptosis inhibitor 5 (API5) were significantly reduced after expression of *REST* in INS-1E cells (Fig. 5B). While IRS2 and PTPRN are known to have anti-apoptotic effects [4], [10], the latter four proteins were not, prior to our study. To verify whether these genes were downregulated *in vivo* upon REST expression, and could account for the apoptotic events occurring in the diabetic RIP-REST mice, we analyzed islets of RIP-REST mice. qPCR experiments showed that the expression levels of several REST target genes, including Gjd2, Mapk8ib1, Ptprn and Cdk5r2 were reduced (Fig 5C). In contrast, we did not see any downregulation for Irs2, Nrg1 or Ctnnd2 in these transgenic mice (Fig 5C). Decreased production of Cdk5r2 upon REST expression was also confirmed at the protein level using INS–1E cells transduced with Ad-REST and Ad-GFP as a control (Fig. 5D). We further evaluated the possibility that beta cells from RIP-REST mice were suffering from apoptosis, due to ER stress that may be induced by enhanced levels of RIP-mediated protein translation. However, we failed to detect any increase in the transcript levels of the ER stress markers, Chop, Bip and splice Xbp-1 in the islets of RIP-REST mice, compared to wild type animals (Fig. S1).

### Specific siRNA silencing of Cdk5r2 increases INS-1E cells susceptibility to cytokines

**Figure 6:**
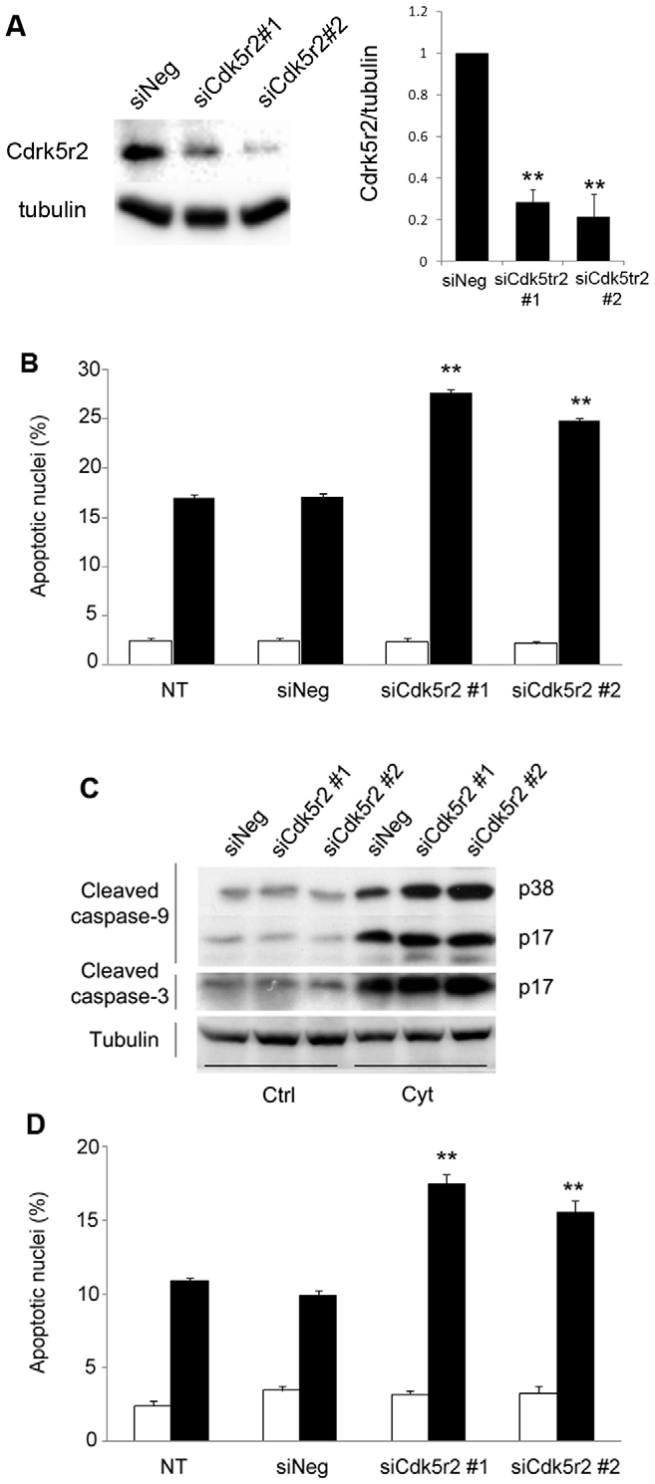
CDK5R2 protects beta cells against cytokines and palmitate. *A*. Left panel, immunoblotting of total proteins from INS-1E cells, 72 h after transfection with a negative siRNA (siNeg) or siRNAs against *Cdk5r2* (siCdk5r2#1 and siCdk5r2#2). Right panel, quantification of the corresponding Cdk5r2 protein levels. Results are mean ± SEM of three independent experiments. **P<0.01 versus values of siNeg-transduced INS-1E cells. *B*. Quantification of apoptotic nuclei in non-transfected INS-1E cells (NT) and INS-1E cells transfected with a negative siRNA (siNeg) or with siRNAs against *Cdk5r2* (siCdk5r2#1 and siCdk5r2#2), treated 24 h with a mix of cytokines (black bars) or not (white bars). Results are mean ± SEM of six independent experiments. **P<0.01 versus controls. *C*. Immunoblotting of total proteins from INS-1E cells transfected with a negative siRNA (siNeg) or siRNAs against *Cdk5r2* (siCdk5r2#1 and siCdk5r2#2), treated 24 h with a mix of cytokines (Cyt) or not (Ctrl). The cleavage of caspase-9 and −3, induced by cytokines, is higher in siCdk5r2- than in siNeg-treated cells. *D*. Quantification of apoptotic nuclei in non-transfected INS-1E cells (NT) and INS-1E cells transfected with a negative siRNA (siNeg) or siRNAs against *Cdk5r2* (siCdk5r2#1 and siCdk5r2#2), exposed 15 h to control medium (white bars) or palmitate (black bars). Results are mean ± SEM of six independent experiments. **P<0.01 versus controls.

We then assessed the role of *Cdk5r2*, given that this protein was not yet known to be involved in beta cell apoptosis. Out of three siRNAs tested, we selected two specific rat *Cdk5r2* siRNAs (siCdk5r2#1 and siCdk5r2#2) which efficiently silenced the target sequence, as judged by qPCR experiments conducted on transfected INS-1E cells (data not shown). Transfection of these siRNAs into INS-1E cells resulted in a 70% to 80% reduction of *Cdk5r2* protein level after 72 h (Fig. 6A). This change did not affect the levels of *Cdk5r1* (also called *p35*), another activator of CDK5 (data not shown). We then evaluated the effect of silencing *Cdk5r2* expression on the capacity of INS-1E cells to survive to a cytotoxic attack or palmitate incubation. To this end, 56 h after siRNA transfection, INS-1E cells were treated during 24 h with a mix of IL1-β, TNF-α and IFN-γ. *Cdk5r2* silencing *per se* had no effect on INS-1E cells viability, as compared to non-transfected cells (Fig. 6B). However, siCdk5r2#1 and siCdk5r2#2 transfection increased the cytokine-induced apoptosis by 60% and 50%, respectively, as compared to cells transfected with the control siRNA (Fig. 6B). Western blotting performed with total proteins from the same experiment confirmed that Cdk5r2 knock-down aggravated the cytokine-induced apoptosis, as quantified with increased cleavage of caspase-9 and −3, an early and late apoptosis marker, respectively (Fig. 6C). Similarly, siCdk5r2#1 and siCdk5r2#2 transfections increased apoptosis induced by a 24 h treatment with palmitate (0.5 mM) by 60% and 40%, respectively (Fig. 6D). These results indicate that *Cdk5r2* contributes to protect beta cell against both cytokines and free fatty acid-induced apoptosis.

## Discussion

Recent bioinformatic studies on the distribution of RE-1 binding sites in the genome, combined with analysis of chromatin occupancy mediated by REST, have revealed the existence of several hundred putative REST target genes [19]–[24]. The caveat of these large output studies is the lack of functional assessment of the candidate genes. The generation of transgenic mice expressing *REST* in beta cells represents a reliable model to identify the importance of RE-1-containing genes in insulin-producing cells [13]. Specifically, analysis of the glucose-intolerant RIP-REST mice led to the identification of several REST target genes which are key to exocytosis of insulin-containing granules [13]. We now document a role for REST target genes in beta cell survival in a RIP-REST mouse line which displayed diabetes, as a result of a high level of *REST* expression, which was associated with a massive loss of adult beta cells.

This massive loss is due to the down-regulation of multiple REST target genes, whose number is likely to increase with REST levels. Indeed, transcription factor abundance is an important parameter influencing the activity of many, though not necessarily all cognate DNA binding motifs [25]. Furthermore, sequence variations in the RE-1 binding motif establish a hierarchy of binding affinity for REST to its target genes, indicating that genes bearing a weakly conserved RE-1 motif have a suboptimal binding affinity for REST [26]. Thus, it is likely that more genes are down-regulated in the diabetic than in the glucose-intolerant RIP-REST transgenic mice, due to the higher expression of *REST* in the former animals. Using RE-1 database screenings and our *in vitro* model of REST gain of function, we further show that REST expression triggers 1) beta cell death under normal conditions 2) increased sensitivity to cytokines and palmitate 3) decreases the expression of several genes coding for known effectors of beta cell survival/proliferation.

REST expression does not impact beta cell proliferation at P2, and our observations suggest that the major consequence of high levels of *REST* expression is a gradual postnatal loss of beta cell mass. In a separate study, using an inducible REST transgene under the control of the Pdx1 promoter, we also observed postnatal beta cell loss leading to hyperglycemia (unpublished data). While the effect of REST expression on INS-1E cells survival was modest in normal conditions, we suggest that the expression of REST target genes is crucial during the postnatal remodeling of primary islet cells [27].

Searching for RE-1-containing genes bearing pro-survival activity, we identified MAPK8IP1 [9], [28], GJD2 [29], IRS2 [4] and PTPRN [10]. We further discovered that *Cdk5r2* [30] is an additional, hitherto disregarded RE-1-containing gene that is essential for beta cell survival. CDK5R2, like CDK5R1, is a neuron and beta cell-specific activator of the atypical kinase CDK5. This kinase phosphorylates a large number of substrates, involved in a variety of neuronal and non-neuronal functions (reviewed in [31]). Conflicting reports have described the role of CDK5 and its activators on insulin secretion. Whereas inhibition of CDK5R1 activity was reported to promote insulin secretion [32], the association of CDK5 to CDK5R2, but not CDK5R1, has also been shown to induce insulin release [33]. The role of CDK5 in neuronal survival is also a matter of debate. Deregulation of CDK5 activity by p25 has been involved in numerous neurodegenerative diseases [34], [35], while Cdk5-loss-of-function studies have revealed its pro-survival function in neurons under normal [36]–[39] or stress conditions [36], [37], [40], [41]. Similarly, it is not clear whether CDK5 plays a prosurvival activity or not in pancreatic beta cells. While its inhibition promoted PDX-1 nuclear localization and increased beta cell survival in response to glucotoxicity [42], a recent publication showed that decreased levels of CDK5 triggered apoptosis via loss of activation of focal adhesion kinase and decreased PI3K/AKT signaling [43]. So far, however, the role of CDK5R2 in beta cell survival has not been explored. In the latter study, the authors proposed that CDK5R1 may be the activator form of CDK5 that is mediating the prosurvival activity, neglecting the role of CDK5R2. Here, we show that *Cdk5r2* belongs to the cluster of RE-1-containing genes, and therefore that it is crucial and specific to neurons and pancreatic beta cells. Moreover, even though the silencing of *Cdk5r2* is not inducing beta cell death under normal conditions, we provide evidence that its knock-down by two distinct siRNAs leads to enhanced sensitivity of INS-1E cells exposed to pro-inflammatory cytokines or palmitate, two mediators of beta cell apoptosis in T1D and T2D, respectively [44].

In summary, the present findings extend our previous observations that ectopic expression of *REST* in beta cells allows for novel insights into the specific traits that make a beta cell what it is [12]. Our work further demonstrates the need for healthy beta cells to express the whole set of RE-1-containing genes. Specifically, we show here that high chronic levels of REST in beta cells leads to diabetes, due to prolonged repression of multiple REST-target genes known to contribute to beta cell survival. We further identify a hitherto neglected anti-apoptotic player, CDK5R2, which we show to be essential for beta cell survival under conditions that trigger apoptosis. Given the large number of estimated REST targets, it is anticipated that several other genes, which remain to be identified and validated, may play an important role in beta cell life and function under the native conditions of lack of REST expression.

## Materials and Methods

### Transgenic mice

Transgenic mice specifically expressing *REST* in beta cells were obtained by pronuclear injection of C57Bl6/J zygotes as previously described [13]. Our institutional review committee for animal experiments approved all the procedures for mice care, surgery and euthanasia.

### Pancreas perfusion and insulin secretion

Mice were anesthetized i.p with 100 mg/kg b:w sodium pentothal, and prepared for pancreas perfusion as previously described [45]. The pancreas was perfused at 1.5 ml/min at 37°C with modified Krebs-Ringer HEPES buffer supplemented with the indicated concentrations of glucose. The pancreatic effluent of the first 30 min of perfusion with basal glucose (1.4 mmol/l) was not collected. After this equilibration period, the effluent was collected in 1-min fractions from a catheter placed in the portal vein. The insulin content of each fraction was determined by radioimmunoassay.

### Immunohistochemistry and confocal microscopy

Pancreas were fixed in 4% (w/v) paraformaldehyde, equilibrated overnight in 15% (w/v) sucrose, embedded in 15% sucrose-15% (w/v) gelatin and quickly frozen in methylbutane/liquid nitrogen. Cryosections were permeabilized for 20 min in 0.1% Triton X-100, then blocked in 1.5% BSA in PBS for 30 min, and incubated overnight at 4°C with either polyclonal rabbit antibodies against human REST [46], glucagon (Dako, Baar, Switzerland, 1/500), proliferating cell nuclear antigen (PCNA, Dako, 1/200) or polyclonal guinea-pig antibodies against insulin (Zymed Lab. Inc., San Francisco, USA, 1/500). Primary antibodies were detected using appropriate fluorescein or rhodamine-conjugated antibodies. A supplementary antigen-retrieval treatment was required for anti-PCNA, which consisted of boiling the slides in citrate buffer (10 mM Na-citrate, pH 6) for 10 min before incubation with the blocking solution. Semi-quantitative immunodetection of REST was performed using secondary antibodies coupled to horseradish peroxidase from the rabbit Vectastain ABC kit, using 3-amino-9-ethylcarbazole (AEC) as substrate, according to the manufacturer's instructions (Vector Lab. Inc., Burlingame, CA, USA). Sections were viewed on either a Leica DM5500 fluorescence microscope or a Leica SP2 upright confocal microscope (Leica, Nidau, Switzerland). TUNEL labeling was performed using the *in situ* cell death detection kit, according to the manufacturer's instructions (Roche Diagnostics, Rotkreuz, Switzerland).

### Image processing and cell mass quantification

For semi-quantitative assessment of REST abundance in beta cells of transgenic mice, images have been processed as follow using the ImageJ software: images were first converted to a 32 bit format and subjected to background substraction. A binary image was then created from a duplicated cropped region of interest (i.e an islet) by applying the MaxEntropy thresholding method. After application of the “open” binary operation, particles were detected fixing the minimum size at 100. The average pixel intensity of nuclei (i.e, identified particles), was then measured from the original cropped image and values obtained from at least 10 islets from three animals for each group were subjected to statistical analyses.

For cell mass quantification, the relative area occupied by the different types of islet cells were measured on 16 sections taken throughout the entire pancreas and separated by at least 200 μm (adult animals), or 150 μm (newborn animals). Islet cells and total pancreas areas were measured using an ACECAD Professional graphic tablet connected to a Quantimet Leica 5001 (Leica, Cambridge Ltd, England) programmed for semiautomatic measurement of areas.

### Glucose tolerance test

Male mice of 12–16 weeks were fasted for 15 h before blood samples were collected from the tail vein at 0 (fasting blood sample), 15, 30 and 120 min after an intraperitoneal injection of glucose (2 g/kg of body weight as a 20% solution). Blood glucose levels were measured with a Glucometer (Bayer AG Health Care, Switzerland).

### Cell line and mouse islet isolation

The rat insulinoma cell line INS-1E was maintained in RPMI 1640 medium, as previously described [13]. For infection experiments, INS-1E cells were seeded in 12-well plates and cultured for 48 h before infection with adenoviruses as previously described [46], with a multiplicity of infection of 2 or 5. Islets of Langerhans of adult C57BL/6 male mice, weighing 25–30 g, were isolated and cultured as previously described [13].

### RNA isolation and real time RT-PCR

RNA isolation was performed as previously described [47]. Quantitative RT-PCR (qPCR) was performed using the SYBR Premix Ex Taq PCR Kit TaKaRa (Axon Lab, Switzerland) in a Lightcycler (Roche Diagnostics GmbH, Mannheim, Germany), as previously described [47]. cDNAs were amplified using specific primers (Table S1).

### Apoptosis assay

Apoptosis was determined by scoring cells displaying picnotic nuclei stained with the DNA-binding dyes Hoechst 33342 (10 μg/ml; Molecular probes, Eugene, Oregon, USA), as visualized under an inverted fluorescence microscope. A minimum of 500 cells were counted in each experimental condition and viability was evaluated by two independent observers, one of them unaware of sample identity.

### Western blotting

Western blots were performed as previously described [47]. Specific protein levels were revealed with polyclonal rabbit antibodies against Cdk5r2, cleaved caspase-3 and cleaved caspase-9 (Cell Signaling Technology, Danvers, MA, USA). Monoclonal antibodies against α-tubulin (Sigma-Aldrich, St-Louis, MO, USA) were used to normalize the signals.

### siRNA design and cell transfection

Specific siRNAs against rat *Cdk5r2* were selected using the siRNA Target Finder (Ambion, Austin, TX, USA). The silencing efficiency of each construct was checked at the transcript level using target genes fused to a luciferase reporter gene, as previously described [13]. Two efficient siRNAs, siCdk5r2#1 (5′-GCCAGCGUCCACCGGUCCCUU-3′) and siCdk5r2#2 (5′-GCCACCGUCGCUAUUACCGUU-3′), and the negative siRNA(5′AGGUAGUGUAAUCGCCUUGUU-3′) were synthesized from Microsynth (Balgach, Switzerland). INS-1E cells were seeded in 12-well plates and incubated overnight in antibiotic-free medium. Negative or siRNA duplexes (60 pmol/ml) targeting *Cdk5r2* were mixed with Lipofectamine2000 reagent according to the manufacturer's instructions (Invitrogen, Basel, Switzerland). siRNA-lipofectamine complexes were added to the cells and incubated for 56 h. Cells were then exposed for 24 h to either a mixture of rat IL-1β (0.5 ng/ml), mouse TNFα (1 ng/ml) and rat IFNγ (10 ng/ml) (R&D Systems, Minneapolis, MN, USA) [48] or to palmitate (sodium salt; Sigma-Aldrich, St-Louis, MO, USA). Palmitate was dissolved in 90% ethanol, heated to 60°C and diluted at a final concentration of 0.4 mM in RPMI 1640 with 1% BSA and 1% fetal calf serum) [49].

### Statistical analysis

Data were expressed as mean ± SD. Differences between means were assessed using ANOVA followed by *t*-tests with Bonferroni correction for multiple comparisons. Statistical significance was defined at a value of *P*<0.05 (*), *P*<0.01 (**) and *P*<0.001 (***).

## Supporting Information

Figure S1 **ER-stress markers are not upregulated in islets of transgenic RIP-REST mice.** qPCR experiments on islets from 5 month-old animals show identical levels of the transcript of ER-stress markers in islets of RIP-REST mice. White bars are wild type mice and black bars, RIP-REST mice.
(TIF)

Table S1 **Specific primers for real time RT-PCR.**
(DOCX)
