# Supplementary figures and images for: Specific Silencing of the REST Target Genes in Insulin-Secreting Cells Uncovers Their Participation in Beta Cell Survival
Source: PLoS One. 2012 Sep 20;7(9):e45844. doi: 10.1371/journal.pone.0045844 (PMC3447792; doi:10.1371/journal.pone.0045844)

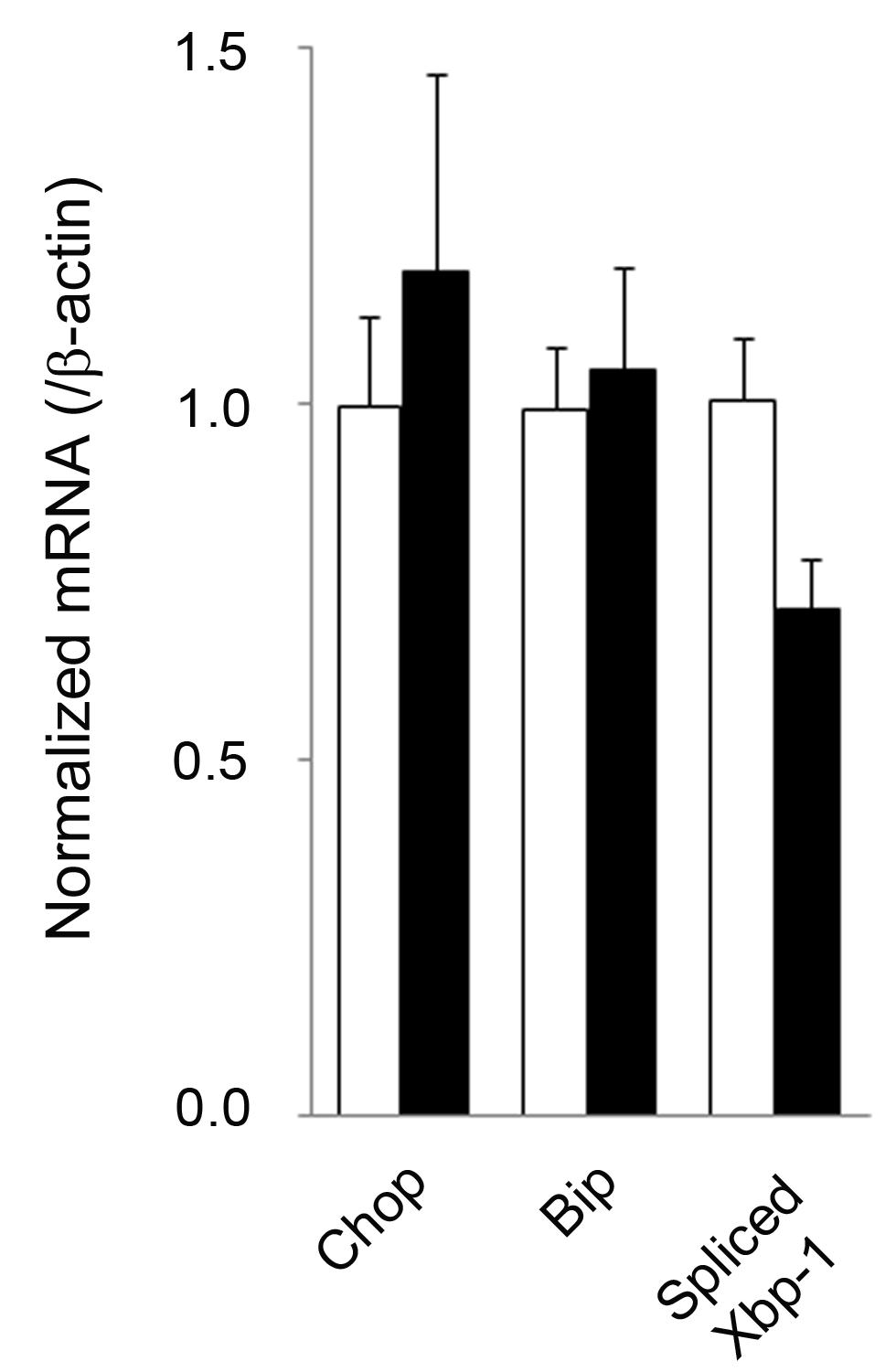

Supplement: Figure S1 — ER-stress markers are not upregulated in islets of transgenic RIP-REST mice. qPCR experiments on islets from 5 month-old animals show identical levels of the transcript of ER-stress markers in islets of RIP-REST mice. White bars are wild type mice and black bars, RIP-REST mice. (TIF) [file pone.0045844.s001.tif]
