# Supplementary material for: Specific Silencing of the REST Target Genes in Insulin-Secreting Cells Uncovers Their Participation in Beta Cell Survival
Source: PLoS One. 2012 Sep 20;7(9):e45844. doi: 10.1371/journal.pone.0045844 (PMC3447792; doi:10.1371/journal.pone.0045844)
Supplement: Table S1 — Specific primers for real time RT-PCR. (DOCX) [file pone.0045844.s002.docx]

**Table S1:** **specific primers for real time RT-PCR**

| **Gene** | **NCBI**  **Access N °** | **Sense primer (5’-3’)** | **Antisense primer (5’-3’)** |
| --- | --- | --- | --- |
| **REST** | NM_011263 | GGGATGTGTCTGGGAAGAAG | CCTGTTTGTCCGTCTGTGTG |
| **REST** | NM_005612 | acacctgaaacaccacacca | AACTTGAGTAAGGACAAAGTTCACA |
| **Cdk5r2** | NM_009872 | TACTCCTACATGGGCAACGA | CTTGAAAGACTTGCGTGAAGA |
| **Cdk5r1** | NM_053891 | AAGGCCACACTGTTTGAGGA | TGCTCTGGTAGCTGCTGTTG |
| **Gjd2** | NM_010290 | ATACAGGTGTGAATGAGGGAGGATG | TGGAGGGTGTTACAGATGAAAGAGG |
| **Mapk8ip1** | NM_001202446 | CGCCCTCTGTTGCTATTCTC | TTTGACGCCTATCTTGACACC |
| **Ptprn** | NM_008985 | GGCTCCTCCTCAGTCCTTCT | AATCTCCAGCAGCCTCACTC |
| **Ptprn** | NM_053881 | TCCCAAGACATCCAGTCCTC | TCTCCAGCAACTTCACTCCA |
| **Irs2** | NM_ 001168633 | ATCCACAGCCAGGAGACAAG | AAGTCGATGCTTGCGTAGGT |
| **Nrg1** | NM_031588 | tctgtatgcccaggaatggt | TGCCAATAGGTTGGAAATGG |
| **Ctnnd2** | NM_001065606 | TGAGTGCTCAAACCCAGACA | GGAGGAGCTCCACAAGAATG |
| **Api5** | NM_007466 | GACAACTTCGCTTGGCTCTC | TTTGCCCAATCTCAACTTTCT |
